# Supplementary material for: Are carbon emissions trading and green financial instruments synergistic? -Comprehensive quantitative research based on content analysis
Source: PLoS One. 2024 Mar 7;19(3):e0298601. doi: 10.1371/journal.pone.0298601 (PMC10919601; doi:10.1371/journal.pone.0298601)
Supplement: S1 Data — (ZIP) [file pone.0298601.s001.zip › Data/地级市Z_F工作报告.zip!/┴╔─■/╡ñ╢1⁄2╩╨/└┤╫╘╣1⁄2╓┌║┼í╛┬φ┐╦╩2╛▌═°í┐_2016.docx]

各位代表：

我代表市政府向大会报告工作，请予审议，并请市政协委员和各位列席人员提出意见和建议。

一、“十二五”时期经济社会发展回顾和2015年重点工作

“十二五”时期，面对复杂严峻的国内外形势，在市委的正确领导下，在市人大、市政协的有力监督和支持下，全市上下深入贯彻落实党中央、国务院和省委、省政府的决策部署，抢抓发展机遇，加快经济结构调整，深化改革开放，采取积极有效措施应对经济下行压力，实现了全市经济平稳发展，社会事业全面进步。

经济实力进一步提升。“十二五”时期，预计全市地区生产总值年均增长7%，人均地区生产总值达到4.16万元；一般公共预算收入累计完成560.1亿元，是“十一五”时期总量的2.3倍；固定资产投资累计完成4070亿元， 是“十一五”时期总量的2.2倍；社会消费品零售总额年均增长12.8%；外贸出口总额年均增长7.6%。

产业结构得到优化。粮食产量实现五连丰，高效特色农业发展格局基本形成，建立起优质稻米等15大特色农产品基地，一批投资10亿元以上农产品加工大项目陆续投产，农产品出口稳居全省前列。中国驰名商标和国家地理标志证明商标总量均达到10件。县域经济实力不断增强，东港市、凤城市先后进入全国百强县行列。工业自主创新能力不断提升，全市有22家企业建立了院士工作站，生物基尼龙56等多个自主创新项目实现开工建设。本钢不锈钢冷轧、老东北谷物大豆深加工等一批重大工业项目相继投产。汽车及零部件、仪器仪表、防护纺织制品、增压器、满族医药及健康产业集群被列入省级产业集群。服务业增加值比重五年间提高了8.1个百分点，万达广场等一批城市综合体相继建成，服务业集聚区功能进一步完善，物流、旅游、金融等现代服务业发展势头良好。

一批重大基础设施项目建设顺利完成。东北东部铁路、沈丹客运专线、丹大快速铁路，丹通、丹海高速公路，中朝鸭绿江公路大桥，丹东港20万吨矿石等多个大型专业化泊位，丹东机场新航站楼等一批重大交通设施项目相继竣工投入使用，城市新建、改造、大修道路210多条，功能齐全、四通八达的现代化综合交通网络基本形成。港口吞吐能力大幅提高，吞吐量是五年前的2.7倍。电力设施不断完善，蒲石河抽水蓄能电站并网发电，三湾水利枢纽及输水工程主体完工，金山热电实现发电和集中联网供热。

对外开放取得积极成果。丹东被列入全国重点开发开放试验区，被海关总署批准成为全国首批出境加工试点地区，被省政府批准成立高新区和全省首个边民互市贸易区。成功举办了4届中朝博览会。丹东新区、大孤山经济区、高新区、翡翠湾等沿江沿海区域实现了大规模开发建设，点线面联动发展，对全市经济辐射带动作用显著增强。东北东部合作有序推进，丹东港在东北东部地区建设的40多个“陆港”相继投入使用，丹东与俄罗斯和蒙古的对外贸易实现海铁联运。

生态环境持续改善。生态建设全面推进，耕地、森林、水资源保护力度加大，环境治理效果显著，资源集约利用水平持续提升。“四城联创”和“宜居乡村”建设成效明显，成功创建国家园林城市，荣获“全国文明城市提名城市”和“省文明城市标兵”称号，国家卫生城创建工作稳步推进，国家环保模范城通过省级考核验收，被评为“中国十佳空气品质城市”。

各项改革不断深化。政府职能转变和简政放权步伐加快，清理行政职权项目516项，商事制度、建设工程审批制度改革成效初步显现，公共行政服务体系不断完善。农林综合改革全面推进。国有企业、财政金融、文化、医药卫生等领域改革进一步深化。

社会事业发展和人民群众生活水平稳步提高。圆满完成了第十二届全运会丹东赛区赛事和第十二届省运会承办任务，全民健身广泛开展。全面完成义务教育“双高”普九任务。公共文化服务能力和供给水平不断提升，文化产业稳步发展。城乡基层医疗服务体系进一步完善，医疗服务水平不断提高。“十二五”期间，城镇、农村常住居民人均可支配收入年均分别增长12%和12.6%。建立了统一的城乡居民基本养老制度，社保扩面任务全面完成。全市城镇实名制就业超过25万人，登记失业率控制在4.2%以内。实现29万农村贫困人口脱贫。累计建成保障房4.2万多套，城镇人均居住面积五年间增加了4.4平方米。实施了菜篮子、饮水安全、老旧小区改造、道路畅通、特殊困难群众关爱等一批民生工程。连续6届荣获“全国双拥模范城”称号。“平安丹东”、“法治丹东”、“诚信丹东”建设全面推进。

各位代表，刚刚过去的2015年，更是我们积极应对经济下行压力、保持经济在合理区间内运行的重要一年。预计全市地区生产总值完成990.5亿元，按可比价格计算，比上年下降3.1%；固定资产投资完成593亿元，下降35%；一般公共预算收入实际完成66.3亿元，下降47.3%；预计社会消费品零售总额完成502.3亿元，增长5%；外贸出口总额完成26亿美元，下降13.8%；城镇居民可支配收入完成24536元，增长7%；农村居民可支配收入完成12335元，增长7%。过去的一年主要做了以下重点工作：

(一)狠抓稳增长各项工作，保持了经济平稳运行

全市确定的119个亿元以上重点调度项目，实现开复工85个。

积极应对严重旱情，开展生产自救，实现了“旱灾不减产”，粮食产量突破百万吨。进一步优化调整种植结构，加快发展现代农业，新建特色精品示范基地8个、省级现代设施农业小区6个，发展软枣猕猴桃、油菜花、洋菇茑等特色农业近2万亩。经济林面积达11.4万亩。落实资金扶持重点蔬菜基地发展。建设国家级水产健康养殖示范场4个，省级规模畜禽生态养殖场10个、标准化养殖场2个。农业产业化规模进一步壮大，6个投资10亿元以上农产品加工大项目完成总投资74%；10个亿元以上农业产业化项目实现开工建设。新增国家级和省级出口示范区各1个，农产品出口额预计实现7亿美元。

加强对工业经济监测、研判和企业的帮扶，出台促进工业经济稳增长的六条意见，开展全市领导干部“进千企解难题促发展”、“科技列车丹东行”等系列活动，帮助企业解决了一批生产经营相关问题，48户停产半停产企业实现复产。市中小企业服务平台集聚服务机构100余家，服务体系初步形成。总投资100亿元的丹东港集团现代化木材加工产业园等20个亿元以上工业项目实现开工建设，恒通石墨等25个亿元以上项目投达产。全市战略性新兴产业比重占规模以上工业的9.1%，企业试成新产品710项，投产360项。满族医药及健康产业集群升级为省级重点产业集群，工艺美术、非金属新材料产业集群正在形成。

加快推动服务业发展。突出旅游业的牵动作用，鸭绿江精品旅游带重点项目控制性规划编制完成，为全力打造鸭绿江国际旅游品牌奠定基础。开通了中韩旅游班列，启动了赴朝鲜新义州登岸游项目。中朝文化展览馆建成开馆。成功举办了第十二届“东博会”暨鸭绿江国际旅游节、观鸟节、桃花节等主题节事活动，实现旅游业的持续火爆，预计全年旅游总收入增长15%；国内和入境旅游人数均增长13%。凤城大梨树村和东港獐岛村分别被国家有关部委授予“全国宜居示范村”、“中国最美休闲乡村”称号。商贸物流业保持快速发展，我市被列为全国66个区域级流通节点城市之一，大东港物流等5个物流产业集聚区日益规范完善。新兴服务业得到快速发展，全市电子商务交易额达12亿元，电子商务产业园有20户电商企业入驻运营，东港市被国家商务部、财政部批准为电子商务进农村综合示范县。通过出台政策、召开房交会、赴外促销等措施，实现了商品房销售额的正增长，保持了房地产市场平稳发展。

财政金融保障作用进一步增强，全年向上争取各类补助资金比上年增加15亿元，一般公共预算支出完成213.2亿元，重点向改善民生、促进发展倾斜。多方筹集资金74亿元，确保了全地区企业退休人员养老金及时足额发放。严格控制新增债务，积极化解存量债务，争取债券置换60亿元，减轻债务利息负担2.5亿元。全市各类金融机构达到139家，实现各类融资253亿元，银行新增贷款连续六年突破百亿元。企业上市呈现良好推进态势，华隆电力在新三板成功挂牌。

(二)注重借势借力，加快发展外向型经济

丹东港开放步伐不断加大。重点建设了多个5至20万吨级散货、通用集装箱、滚装等专业化泊位和20万吨级航道及配套基础设施；加快推进丹东港至俄罗斯和蒙古物流通道建设。港口货物吞吐量预计达到1.5亿吨，增长8.9%。临港产业中，老东北农牧公司30万吨稻谷加工厂建成投产，大豆菜籽等油脂压榨项目进行车间主体施工和设备安装；木材物流园区基础设施建设完工，绥芬河广达木业等企业入驻生产；海洋工程装备园正在建造耙吸式挖泥船等海工船只以及配套泵船，3万吨级船坞正在进行设备安装、调试。

重点开发开放工作取得实质性进展。国门湾中朝边民互市贸易区正式启动，填补了全省边民互市贸易的空白；

《丹东重点开发开放试验区实施方案》编制完成并上报国家待批；丹东机场临时开放获得批准， 开通了丹东至首尔的临时客运包机；积极推进对朝出境加工试点地区建设，有5家企业获准出境加工业务资质；第四届中朝博览会上升为国家级展会，内容更加丰富，形式更加多样，总交易额达1.5亿元，参会人数达28万人次，展会的知名度和影响力不断扩大。通过加大政府补贴、开拓新兴市场、引入信用保险、对外贸企业帮扶等措施，积极应对地缘政治对我市外贸出口的冲击。深入开展国际合作，批准境外投资项目8个，丹东港、曙光、宝华、金丸集团等企业境外投资得到实质性推进。《东北东部经济带发展规划》纳入国家“十三五”规划，东北东部城市间经贸、物流、旅游等方面合作进一步加强。

新区等经济区建设稳步推进。丹东新区钟表珠宝产业园、纳诺钢结构制造二期等重大项目开工建设，交通、教育、医疗等配套设施不断完善。大孤山经济区基础设施建设项目陆续进入收尾阶段，“三纵两横”路网、园区配套设施、大洋河14万吨应急供水、大鹿岛客货码头等17个重点项目当年完成投资约6亿元；精密加工园区入驻项目31个。高新区起步区道路、管网等配套设施施工完毕，生物基尼龙56项目一期工程完成设备安装；9个亿元以上项目正在同步建设；13个项目入驻育成中心。翡翠湾实现了德丰时代广场、柏林大道等一批项目开工建设。

有针对性地开展招商引资工作。围绕旅游等传统产业及电子商务等新兴产业，开展“点对点”招商、以商招商，促成中国供销集团辽东农产品电商物流、王老吉系列饮料生产等一批大项目签约落地，全年共引进内资到位资金350亿元。

(三)加大基础设施建设力度，强化对经济的拉动作用

备受期待的沈丹客专和丹大快铁相继开通，沈阳、大连对丹东的辐射和带动作用进一步增强，仅去年“十一”期间，铁路抵丹乘客就同比增长174%，对我市旅游、物流等服务业以及经济社会发展产生了深远影响。

结合省“绿叶杯”竞赛活动，实施了大规模城市基础设施建设、改造和卫生环境整治。我市第一个PPP(政府和社会资本合作)项目—同兴垃圾场改造工程已经启动，正在进行招投标。粪便无害化处理厂竣工投入使用。花园路、金山大街和东平大街三个城市道路出口拓宽改造完成，金山立交桥和四号干线锦江山隧道工程主体完工，大众路、曙光路、三纬路等21条道路大修和26条道路维修全部实施完毕，对六纬路、七经街等城市主干道进行提升改造。花园河、大沙河城区段治理工程加快实施。三湾水利城市应急供水工程实现管道通水，四道沟水厂完成调试。大连至丹东“西气东输”项目先期工程开工建设。

连接中朝鸭绿江公路大桥与丹大、沈丹高速公路的新丹线建成通车。大东港疏港高速公路开工建设。鹤大高速前阳出口项目得到批复，征地工作基本完成。宽甸至本溪碱厂高速公路项目纳入国家高速公路网规划。

以“大禹杯”创建和“宜居乡村”建设为载体，加大农村基础设施建设。全市投入4亿多元资金进行乡村环境集中整治，实施了一批道路硬化、亮化美化绿化、垃圾污水处理、环卫设施改造等工程，累计改造农村无害化卫生厕所6万多座，改造农村危房1600多户，有效地改善了乡村居民生产生活环境。投入3亿多元，用于农田水利设施、农村饮水安全工程建设和水库移民后期扶持，极大提高了全市农建水平，其中建成农村饮水安全工程4400多处，基本解决了农村人口的饮水安全问题。

(四)深化各项改革，激发发展内生动力

加快审批制度改革，制定出台了我市《关于进一步优化建设工程项目审批流程实施方案(试行)》、《建设工程项目审批流程图》，使建设项目审批流程得到极大优化，企业审批成本减少约80%。全年取消和下放行政审批项目76项、其他行政职权22项，前置改后置审批项目58项。实行权力清单、责任清单制度，确定市直43个部门保留职权事项2108项，精简比例达49.7%。实施“三证合一”企业登记制度改革，全市工商系统新登记各类市场主体比上年增长18%。认真贯彻落实新《预算法》和“三公经费”公开，全市共有5000多户企业纳入“营改增”改革。实施驻丹央企供水、供电、供暖和物业管理分离移交。稳妥推进农村土地确权工作，全面完成省政府下达的115万亩确权任务，东港市实现了“整县推进”。县级公立医院改革全面启动，并纳入取消药品加成试点；卫生计生整合改革完成，每个乡(镇)都设立了公办卫生院。在全省率先推行食品药品安全监管“一乡一所”建制，乡镇和行政村分别设立了派出机构和协管员，监管效能进一步提高。丹东出口联检部门在辽宁地级市中率先启动了“信息互换、监管互认、执法互助”工作，通关效率提升50%。

(五)深入实施民生工程，推动社会事业全面发展

加大民生工作力度。通过举办各类高端、专场及常规招聘会、征集高校毕业生就业岗位、强化普惠制培训等措施，推动大众创业、万众创新，增加就业，全年城镇实名制就业5万人，登记失业率控制在4.2%以内。完成了省政府下达的社会保险扩面征缴任务，养老保险、城乡低保、农村五保户对象、新农合、医疗保险补助、取暖救助等标准均有提高。“兴边富民”行动和少数民族发展“百千万户”惠民工程得到深入实施。通过落实产业扶贫、加大机关事业单位扶贫力度等措施，实现1.7万农村人口脱贫。为3500多名贫困残疾人适配了辅助器具，为900多户贫困残疾人家庭实施了无障碍改造。通过深入持续开展城乡环境卫生综合整治、实行市级领导包街路等措施，路边烧烤、马路市场、马路工厂等市容顽疾得到有效遏制，群众生活环境有了较大改善。完成了20个1万平方米以上老旧小区综合整治任务。加大棚改工作力度，对棚户区改造项目实行专题例会，采取快速审批和一事一议的方式，并将商品房销售与棚户区改造有机结合起来，建设棚改房5884套。最好金龟换酒全集

推动社会事业全面发展。公办和普惠制幼儿园在园人数稳步提升，东港市、振兴区、元宝区、合作区通过国家义务教育均衡发展县(市)区督导评估，素质教育工程扎实推进，职业教育加快发展。公共文化服务水平不断提升，社区书屋实现了全覆盖，文化宫等文化设施改造完工。一批多功能健身场建成投入使用，乡镇及村屯体育健身器材实现了全覆盖；组织开展了鸭绿江国际马拉松赛、徒步节、广场舞大赛等一系列全民健身活动。基本公共卫生服务水平进一步提高，并向均等化、专业化方向发展。加强食品安全综合治理，推行食用农产品准入制度。社区居委会换届选举工作顺利完成。

重视生态环境建设。“四城联创”活动深入开展，我市在全省文明城市测评中连续三年名列三甲，顺利通过省卫生城复核验收。大力实施碧水、青山、蓝天工程，积极推进国家生态市创建，成为国家级水生态文明建设试点市，宽甸成为国家首批“生态保护和建设示范区”，大孤山国家级自然保护区湿地修复项目基本完成，凤城草河国家湿地公园项目通过国家专家组评审，近海海域环境质量处于全省最佳水平，全市生态环境进一步改善。

努力维护边境地区平安稳定。加强立体化治安防控体系建设，对各类犯罪保持高压严打态势。制定出台了我市《关于进一步加强边海防工作的实施意见》和《关于进一步加强船舶管理的工作意见》，开展了全市边海防综合整治、伏季休渔专项整治等系列活动，集中拆解“三无”渔船13艘，边海防管控得到加强。全面落实安全生产“党政同责、一岗双责、失职追责”制度，安全生产形势稳定向好。强化信访改革和信息系统建设，信访总量大幅下降。应急管理体系进一步完善。民兵预备役和双拥工作取得新成绩。“平安丹东”建设成效明显。(六)扎实开展“三严三实”专题教育，促进政府自身建设不断加强

严格按照“三严三实”基本要求，持续开展政风行风专项治理。严格执行公务调研、会议文件、公务接待等方面的制度规定，大力整治公务用车、办公用房、因公出国等方面存在的问题。有54人因违反中央八项规定和行政不作为、乱作为而受到党纪政纪处分。

法治政府建设和依法行政全面实施，普法和依法治理工作扎实推进，行政执法水平得到提升。

自觉接受市人大及其常委会依法监督和市政协民主监督，认真听取社会各界和人民群众意见。办理市人大代表建议、政协提案619件，按期办结率、见面答复率、满意率和基本满意率均达到100%。政务公开全面开展。行政监察和审计监督得到强化。

外事、侨务、台务、民族宗教、防震减灾、老龄等工作也取得了新成果。

各位代表，回首丹东“十二五”期间和去年的工作成绩，我们深深地感到，这是省委、省政府和市委正确领导的结果，是市人大、市政协大力支持的结果，也是政府几届班子带领广大人民群众攻坚克难、共同努力的结果。在此，我代表市政府向全市各族人民，向各民主党派、工商联、各人民团体、各界人士以及驻丹单位和驻军部队表示衷心的感谢并致以崇高的敬意!

在总结回顾成绩的同时，我们也清醒地认识到，当前全市经济社会还面临着一些困难和问题，主要有：受整体宏观经济形势影响，经济下行压力持续加大，主要经济指标增长乏力；经济总量仍然相对偏小，与省内外沿海城市比始终未能实现大幅增长，产业结构不够合理，自主创新能力还不强；沿江沿海经济带发展不均衡，新开发区域推进不快；作为我市主导产业和资源丰富的旅游业一直未能做大做强，面对节假日和季节性增加的人流、物流，旅游设施和服务保障能力还显薄弱；财政刚性支出不断增加，债务风险和社保压力进一步加大，财政运行困难；政府系统干部发展激情、担当开拓精神不足，不作为、慢作为现象依然存在。这些问题和不足将在以后的工作中，采取切实有效的措施加以解决。

二、“十三五”时期经济社会发展目标和2016年重点工作

各位代表，“十三五”时期，我国仍处于大有作为的重要战略机遇期，随着经济发展方式的加快转变，新的增长动力正在孕育形成，经济长期向好的基本面没有改变。从我市来看，尽管目前经济下行趋势仍将延续，发展压力依然很大，但“十三五”时期面临着新一轮东北振兴、“一带一路”加快推进、沿边地区开发开放和东北东部经济带崛起等诸多重大发展机遇，为我们进一步深化改革、扩大开放、加快发展经济提供了支撑和保障，也为我们全面建成小康社会提供了信心和勇气。

“十三五”时期全市经济社会总体发展思路是：深入贯彻习近平总书记系列重要讲话精神，以落实“四个全面”战略布局为统领，以“五大发展理念”为指针，以“四个着力”为根本遵循，以“四个驱动”为引擎，坚持以开放促发展、以改革谋振兴、坚决破除体制机制障碍，加快推动产业转型升级，努力提高经济发展质量和效益，全力打造东北东部出海主通道、区域性物流中心、临港产业基地、东北振兴沿边开发开放先导区，进一步提升丹东生态、宜居、旅游、港口城市形象，如期实现全面建成小康社会的奋斗目标。

“十三五”时期，我市地区生产总值年均增长7.3%，人均地区生产总值达到5.7万元，我们将：

把丹东建设成一个城乡社会协调发展、经济实力显著增强的小康城市。到2020年，实现地区生产总值和城乡居民人均收入比2010年翻一番，经济发展质量和效益明显提高，三次产业协调发展，比重调整为10：42：48，结构更加优化。社会保障体系趋于完善。大众创业、万众创新成效显著。实现城乡发展一体化和基本公共服务均等化，居民主要健康指标达到或超过全省平均水平。新型城镇化建设得到大力推进，力争城市棚户区和农村危房改造全部完成，通过“六个精准”和“五项帮扶”等措施，确保农村贫困人口全部脱贫。常住人口城镇化率达到68%，户籍人口城镇化率达到50%。

把丹东建设成一个全方位、宽领域、多层次的开放城市。到2020年，全市开放型经济综合实力不断壮大，全面对外开放的环境和体制机制基本形成。力争建成中朝经贸合作区、中韩经贸试验区。新区城市功能基本完善，大孤山、高新区等经济区达到一定规模。中朝边民互市贸易区贸易点扩大至3—4个；出境加工园区、综合保税区建设初见成效。外贸出口总额实现翻番。

把丹东建设成一个东北东部乃至东北亚现代化港口城市。以丹东港为支撑，以中朝鸭绿江公路大桥、东北东部铁路、中韩日等国际海运航线为载体，对接“辽满欧”、“辽蒙欧”、“辽海欧”大通道，积极建设“一带一路”东北线经济走廊战略节点城市。到2020年，丹东港港口功能进一步提升和完善，货物吞吐量力争达到2.5亿吨。临港产业辐射和带动城市发展的能力明显提高，临港物流功能进一步完善，现代综合物流体系得以建立。加快推动丹东港海洋红港区泊位和配套设施建设，使之成为港口经济新的增长点。

把丹东建设成一个生态、宜居滨城。到2020年，全市资源利用效率明显提升，可持续发展能力进一步提高。森林覆盖率提高到67%，城市生活垃圾无害化处理率达到95%以上，污水集中处理率达到95%以上。空气质量优良天数达到82%以上。创建成为全国文明城市、国家环保模范城市和国家卫生城市。方便快捷的智能公交系统基本建成，新老城区联动发展。成为国内赴朝边境旅游的集散地、东北亚区域观光旅游目的地，跨入全国旅游强市行列。

把丹东建设成一个法治、文明、繁荣的平安城市。各项改革任务不断推进，法治环境、诚信环境进一步改善，社会主义核心价值观更加深入人心，城市文明指数明显提高。“智慧城市”建设得到推进，“平安丹东”建设卓有成效，形成边境地区和谐稳定、安居乐业的社会环境。

丹东“十三五”发展的蓝图已经绘就，我们有理由相信，只要我们勇于抢抓机遇，埋头苦干，未来的五年，一定是全市经济社会事业持续发展的五年，一定是硕果累累的五年!我们的目标一定能够实现!

各位代表，今年是实施“十三五”规划的第一年，全市经济社会发展的主要预期指标是：地区生产总值比上年增长6%；固定资产投资增长6%；一般公共预算收入增长2%；社会消费品零售总额增长7%；外贸出口总额增长5%；城镇常住居民人均可支配收入增长7%；农村常住居民人均可支配收入增长7%。为实现上述目标和任务，我们将全力抓好以下重点工作。

(一)开工一批重大基础设施项目，为经济社会加快发展创造条件

开工建设四号干线爱民街至宝山大街贯通工程，形成城市北部道路环线。

通过PPP模式改造站前广场，实现城市公交、旅游班车和长途客运分流，进一步改善交通秩序。

开工建设鹤大高速前阳出口项目，助推前阳开发区加快发展。

对浪东线疏港铁路进行立交改造，畅通城市交通，满足港口运输需要。

实施东港高铁站周边综合开发项目，年内加快配套基础设施建设，完成站前大道和站前广场工程，开工建设高铁大街和海关北路。

开工建设三湾水库向东港供水工程。

启动鸭绿江取水口上移工程，解决取水量不足和潜在的排污问题。

建设完成大沙河、花园河城市内河治理工程，形成新的景观带。

启动并建设完成新区供电线路工程，满足临港产业和重大装备制造业发展需要。

实施大东沟中心渔港、前阳一级渔港建设，为规范渔船管理和安全生产提供条件。

积极推动爱河抽水蓄能电站项目前期工作开展。金庸小说全集

做好大洋河丹东段综合治理工程前期准备工作。

(二)实施一批产业项目，为经济发展提供后劲

全年计划实施亿元以上项目258个，其中新开工亿元以上项目86个。

实现总投资25亿元的东北亚食品产业园、总投资10亿元的忠世公司油井管、总投资10亿元的采油装备制造、总投资3亿元的百岁山矿泉水生产、总投资3亿元的硼海镇光伏发电、总投资2亿元的中子发生器等一批工业项目开工建设。

实现总投资23亿元的爱民街棚改、总投资18亿元的铁矿沟棚改项目，总投资15亿元的凤城辽东农特产电商物流商贸城、总投资5亿元的蔬菜批发市场移地新建等一批服务业项目开工建设。

实现总投资3亿元的凤城草河国家湿地生态公园、总投资2亿元的宽甸禾丰牧业肉鸡精深加工等一批农林项目开工建设。

为各类项目实施提供保障。严格执行《关于进一步优化建设工程项目审批流程实施方案(试行)》以及《建设工程项目审批中不作为不担当责任追究暂行办法》，大力推行重点项目包保责任制、并联审批制、审批手续代办制、绿色通道等落地推进机制；建立重大项目挂牌督办、项目倒排工期等督办机制；通过定期开展项目推进会和现场调度会，集中力量解决审批环节的瓶颈问题，进一步提高项目开工率、履约率、资金到位率和投产达产率。

加大项目招商力度。依托汽车及零部件、仪器仪表、纺织服装等主导产业，积极组织开展对日韩、香港、台湾，以及京津冀、珠三角、长三角等国家和地区的招商活动。围绕现代木材产业园、满族医药、黄金珠宝等项目开展上下游产业链招商。结合旅游产业定位，强化温泉、旅游综合项目招商。重视项目库建设，认真谋划、筛选、储备一批符合国家产业政策的重大项目，力争项目库中达到可研深度的亿元以上项目超过150个。

(三)加快港口和临港产业发展，促进港口经济结构基本形成

推进丹东港基础设施项目建设，完善港口功能。重点推进10至20万吨级大型专业化泊位、20万吨级航道及防波堤、工作船泊位建设，加快围填海、疏港道路、仓储设施以及港区综合附属设施建设。采取节能环保措施，实现港区降能减污综合改造，打造“绿色港口”。

抓好临港产业，加强与城市联动发展。农业项目中，继续推进大豆菜籽等油脂压榨项目，做好小麦、玉米、饲料等粮食加工项目前期准备。海洋工程装备项目中，力争开工建造引航船等海工船舶20余艘。现代木材产业项目中，在加快建设木材物流园区同时，分期建设年熏蒸处理能力400万立方米的进口木材检疫熏蒸处理区，逐步形成木材进口、加工、物流、仓储一体化产业链。冷链物流项目中，重点实施冷链物流工程，建设低温冷藏库等设施；依托铁路、水运及其他冷链运输力量，形成每年160万吨的配送能力。

加强海外合作，进一步扩大港口开放。重点推进中俄陆海物流通道、中俄石化产业园、俄罗斯森林采伐及木材深加工、中俄船舶制造合作区建设；着力打造中蒙陆海物流基地、临港产业基地加工及中蒙跨境产业合作区等中蒙合作平台。

加快“智慧港口”建设，实现港口管理智能化和物流信息的实时交互。以“互联网+临港产业”为载体，促进互联网与期货、生产、贸易、物流配送有效整合，进一步提升临港产业现代化管理水平，促进临港工业、临港物流增收。

(四)坚持产业结构调整，实现发展速度与质量并重

加快发展现代农业。保障粮食安全，加快菜篮子工程建设，切实提高城区蔬菜供应能力。进一步优化农业种植结构，调减玉米种植面积9.5万亩，集中力量建设食用菌、山野菜、特色水果、中药材等精品农业示范基地。大力开展农产品深加工，积极培育农业产业化龙头企业，实现粮食流通产业园等8个亿元以上农业项目开工建设。推动农业科技创新。保障农产品安全。加快特色蔬菜、板栗、中药材等出口示范基地建设，力争今年新增国家级出口示范基地1个、省级1个。

促进工业企业创新和产品换代升级。制定《中国制造2025丹东实施方案》。加快新能源汽车、智能仪器仪表、新材料、节能环保、电子信息等战略新兴产业发展。积极帮助我市企业与国内知名专家、著名高校和科研院所对接，抓好百项重大技术改造，百户国家、省、市级创新示范企业培育，百项重点新产品试制投产。推动曙光集团新能源汽车基地、东发集团智能化高端燃气表、思凯公司智能计量等高新科技项目建设。继续组织各级、各部门领导干部主动走进企业，采取多种方式帮助企业解难题，有效落实《促进全市工业经济稳增长的若干措施》，进一步完善各类中小企业服务平台。重点推进省级产业集群发展。

大力实施现代服务业。支持电子商务业发展，构建电子商务服务体系，积极培育电子商务示范企业；推进电子商务产业园(基地)建设，创建地域特色农产品和智慧旅游电子商务平台；开展电子商务进农村工作。加快金融业发展，全年实现各类融资210亿元，新增或引进金融机构2家。利用我市得天独厚的气候、旅游、温泉资源优势，注重医养结合，推动宇龙幸福源、北国之春养老社区、东汤圣泉谷温泉新城等一批大型休闲养老地产项目开工建设。通过以棚改带动、推行货币化补偿、政府购买存量房等方式，积极化解房地产库存，稳定房地产市场。加快各类商品市场转型升级，推动商贸物流业向标准化、信息化、智能化、集约化方向发展。进一步繁荣夜经济。结合各类节庆活动，促进会议、展览、旅游市场一体化开发。(五)发挥鸭绿江精品旅游带项目的牵动作用，推动旅游业做大做强

实现宗裕裕龙金汤水世界、北黄海海水温泉度假村等温泉项目投入使用。

开工建设浪头港综合旅游项目，打造观光游览、文化艺术、特色餐饮、科普教育多位一体的国际旅游休闲港。

开工建设珍珠岛旅游项目，突出温泉、休闲度假等特色，打造东北东部高端城市生态休闲岛屿。

开工建设虎山长城系列旅游项目，打造中国长城文化主题公园、长城世界文化遗产扩充景区、辽东边陲古镇风情园。

开工建设东港合隆龙山体育旅游公园项目。

积极推进甲午海战遗址博物馆，凤凰山大唐文化体验城，大孤山明清一条街，东港观鸟园、凤城蒲石河、宽甸黄椅山改造等旅游项目建设。

重点办好鸭绿江口湿地观鸟节、河口桃花节、大孤山庙会、凤凰山庙会、鸭绿江国际啤酒节、大梨树采摘节、天桥沟枫叶节、东港海鲜节、温泉冰雪节等活动，实现旅游节事全年通。

全力抓好各项旅游保障措施。做好全市旅游总体规划及温泉、沟域、乡村旅游专项规划。加快推进凤凰山等景区创建5A级景区。以沈丹客专、丹大快铁以及首尔、杭州、哈尔滨空中航线开通为契机，加强旅游基础设施建设，妥善做好涌入丹东游客的承接服务，确保餐饮住宿、交通疏导、车辆停放、景区安全等各项工作落实到位；进一步开辟对朝旅游项目，拓宽与东北东部、长三角、珠三角、京津冀等区域旅游合作；通过央视一套、旅游卫视、北京繁华区域广告牌匾、网络等平台进一步扩大丹东旅游宣传。加强旅游文化建设，注重挖掘鸭绿江文化内涵和丹东人文历史元素，陆续推出具有地方特色的旅游商品。

(六)抢抓机遇，进一步扩大对外开放

加快对外开放平台建设。进一步完善国门湾边民互市贸易区的基础设施和监管设施，确保年内正式投入运营。着手规划设立出境加工园区。完成丹东综合保税区规划、选址和可行性研究，并启动申报工作。增加丹东机场国内国际航线，力争将机场开放纳入国家“十三五”规划。

努力促进对外贸易企稳回升。结合我市产业发展特点，重点培育纺织服装、汽车及零部件、仪器仪表、电脑软件、木材、水(海)产品、果菜等出口基地；加快转变外贸增长方式，大力支持自主知识产权、自主品牌产品出口；争取一批跨境电子商务平台上线运营；引进一批外贸综合服务企业，拓展新的出口模式。筹办首届中韩(丹东)国际博览会，举办好第五届中朝博览会。

积极参与和融入“一带一路”战略。推进曙光集团投资塞尔维亚整车生产、东方测控集团投资澳大利亚仪器仪表、金丸集团投资埃塞俄比亚全自动胶囊生产线、华纳置业公司投资纳米比亚纳中商贸园区等项目尽快实施。

加强区域合作。借助东北东部铁路、沈丹客专和丹大快铁，进一步推动与东北东部、沈阳、大连的深度融合，扩大发展腹地，主动接受沈阳、大连的辐射。

(七)进一步繁荣新区等重点经济区，实现沿江沿海经济带联动发展

进一步推动新区项目入驻和功能完善，确保钟表珠宝产业园、黄海新能源汽车等项目投产见效；推动高速食品罐、智能电气设备、新区污水处理厂、星海湾城市综合体、浙商大厦等项目开工建设。全面提升新区精细化管理水平，促进商贸、交通、医疗卫生、教育等配套设施进一步完善，提高新区入住率。

加快大孤山经济区“三纵两横”路网收尾、大洋河应急供水项目建设，天然气管道工程争取完成主管线铺设和分输站主体工程，继续做好服装产业园、金利冶金、约翰森汽车配件等重点项目的推进工作。

全力保障高新区生物基尼龙56项目产能达到2万吨，以此为基础做好防护纺织产业链的招商；加快推进智能养老机器人、满族医药等项目建设，加大东北亚食品产业园招商引资力度；依托中科院育成中心积极帮扶企业开展科技研发合作。

稳步推进翡翠湾开发建设，强化土地收储和招商引资，确保落地项目尽快竣工投入使用。

(八)加大工作力度，确保完成各项改革任务

实行政府部门权力清单、责任清单制度，向社会公开公示权力运行图；清理行政职权项目，继续推进行政审批项目取消和下放工作；加强对取消下放审批项目的督促检查，遏制因审批职权取消而放弃监管、因审批职权承接不到位而产生权力真空以及审批环节不减反增情况的发生。

推进市辖区公共行政服务中心建设，进一步完善市、县(市)、乡镇(街道)三级公共行政服务体系；优化网上行政审批平台；整合优化12345政府服务热线。

加快构筑统一的公共资源交易平台，提高公共资源配置效率和效益。

探索建立国企改革优惠政策清单，通过招商引资，推动毛绢厂、纺织厂、国资化纤等壳企业和困难企业改革；实行经营性国有资产统一监管，年底前基本完成市直机关所属企业脱钩改制任务；结合驻丹央企“三供一业”分离移交，促进省属及市属企业社会化职能分离。

稳步推进财税体制改革，继续实施“营改增”、消费税等结构性减税政策。积极争取西部财政政策，拓宽财力来源渠道，提高财政保障能力。进一步做好债券置换工作，探索利用PPP合作模式置换存量债务，化解政府债务风险。

抓好农村综合改革，做好农村土地承包经营权确权登记办证试点工作，建立全方位信息化管理平台，以及县(市)、乡(镇)土地承包经营权流转市场体系。

加快医药卫生体制改革，统筹推进医疗服务和保障、药品供应等综合改革，逐步建立全民医保制度和分级诊疗制度。鼓励、引导社会力量参与公立医院改革。

稳妥推进行政类、生产经营类事业单位改革及机关事业单位分配制度改革。完成公车改革任务。

整合现有资源，建立高效快捷的港口、口岸、机场等管理体制和服务机制。

(九)全力做好民生工作，进一步改善群众生活条件

社会保障工程。完成省下达的“五个险种”扩面征缴任务。确保城镇实名制就业达到4万人、登记失业率控制在4.5%以内。继续提高城乡低保等标准。加强资金统筹调度，确保养老金及时足额发放。

畅通工程。改造东升路、兴四路、春五路、滨江路、十一经街等25条道路及两侧人行步道。继续增加城市停车位。力争解决鹏欣水游城区域交通梗阻问题。

市政工程。有序实施市区燃气、自来水管网改造，确保市政设施安全。开工建设三湾输水工程金山净水厂和山上街供水管线；进一步提升供水质量，实施汤山城供水改造和爱河泵站改造。推进海绵城市建设，对振八街、兴五路、后聚宝街等城市易涝区排水系统进行防涝改造。

安居工程。加大城市北部地区棚户区改造力度，全市新开工建设保障房7000套。对20至30个老旧小区的道路、绿化设施等进行综合整治。持续推进城市集中供热，对88万平方米既有建筑进行为期两年的供热计量和节能改造，进一步提升供暖质量。

扶贫工程。落实精准扶贫，充分发挥驻村工作队作用，建立“一对一”帮扶机制，打好攻坚战；加大基础设施、公共服务设施建设和致富产业投入，大力发展集体经济，实施脱贫致富“整村推进”，确保1.5万农村贫困人口脱贫。

生态环境工程。以“四城联创”和“绿叶杯”竞赛活动为载体，继续实施城乡环境综合整治。推进建立城乡统一的建设用地市场，全面实施不动产统一登记。加强对在建项目的监督检查，加大对违法建筑的查处力度。实现国家卫生城市所有指标全面达标，积极开展“绿色社区”建设等国家环保模范城市创建活动，在全国文明城市测评中继续取得好成绩。大力推进碧水、青山、蓝天和沃土工程，力争年内全部淘汰老旧低效锅炉和黄标车，确保市区环境空气质量优良天数大于300天；加大水源地生态建设与保护力度，确保鸭绿江及其主要支流水质优良，城市集中式饮用水水源水质达标率100%。进一步扩大道路、公园绿地面积，启动环城绿化带建设，提高城市绿化、亮化、美化水平。

(十)加强和创新社会管理，推进社会事业全面发展

全面实施第二期学前教育“三年行动计划”，加快公办和普惠制幼儿园建设；继续推进义务教育均衡发展县(市)区创建工作，实施乡村教师支持计划，全面提升义务教育办学水平；加大教育信息化“三通两平台”建设；大力发展职业教育，创新职业教育人才培养模式，加快构建终身教育体系；实施学校危房改造工程，力争四幼，金汤、六纬路、经山小学和第七中学新建或扩建工程完成。星辰变小说

发挥我市高校优势，有针对性地培养人才；完善激励机制，加大高端人才的引进力度。

加快体育产业发展，建立覆盖城乡的公共体育服务体系，推进社区(乡镇)多功能运动场和城区“笼式多功能场”建设；完善锦江山至元宝山绿色健身步道；建设并免费开放10处示范性城市社区全民健身中心。

全力打造鸭绿江流域文化产业经济带，实施文化惠民、艺术提升、城市文化塑造、城市记忆保护四大工程。加快推进抗美援朝纪念馆改扩建工程。

建立完善市、县、乡、村四级食品药品安全监管体系，进一步提升公众对食品药品安全的满意度。

推进医疗卫生资源优化和服务提升，提高新农合保障能力，将三个县(市)新农合纳入市级统筹范围；加强乡镇卫生院、村卫生室建设，村卫生室实现基本药物制度全覆盖，强化对基本药物制度实施情况的监督；建立人口健康信息管理平台，加强重大疾病防控；大力发展中医药事业；推动全面两孩政策稳妥、有序实施。

继续推进市、县两级信用数据征集平台建设；促进信用产品社会化应用；完善企业信用奖惩联动机制。

全面完成我市第六次全国农业普查各项工作。

开展社会化普法工作，积极做好农民工、下岗失业人员、妇女、未成年人、残疾人等群体的法律援助。狠抓立体化社会治安防控体系建设和常态化打击整治。围绕推进军民深度融合加强边海防建设，加大综合管控力度，强化党政军警民“五位一体”管边控海新机制，严防发生敏感水域捕捞等有影响的涉边案(事)件。不断加强民族宗教工作，积极扶持边境、少数民族地区加快发展。切实维护国家安全。加强国防动员、国防教育和民兵预备役工作。进一步促进军政军民团结，广泛深入开展双拥活动。高度重视安全生产，进一步强化责任落实。深入推进信访工作制度改革，不断畅通和拓宽信访渠道，切实解决好人民群众合理信访诉求。

各位代表，面对“十三五”发展的宏伟目标，各级政府责任重大，使命光荣，任务艰巨。为此，我们将保持昂扬的斗志，拿出“不达目的决不罢休”的韧劲和“抓铁有痕、踏石留印”的实劲，坚定信心、迎难而上，坚决完成各项工作任务。

我们将坚持依法行政，按照依法治市的要求，用法治思维和法治方式推动发展，加快建设法治政府，推进政府机构、职能、权限、程序、责任法定化，做到法无授权不可为、法定职责必须为。切实做好政府立法工作，履行好政府规章制定权。坚持完善重大行政决策法定程序，以及责任追究制度和倒查机制，提升科学、民主、依法决策水平。

我们将坚持把权力运行在阳光下，自觉接受市人大及其常委会的依法监督，接受市政协的民主监督，认真听取人大代表、政协委员、民主党派、工商联、无党派人士和各人民团体的意见。进一步强化监察、审计等专门监督。全面推行政务公开，力争所有工作都接受人民的监督，充分体现人民的意愿。

我们将坚持改革创新，力争变中求新、变中求进、变中突破，“加减乘除”一起做，扬长避短发挥比较优势，走出一条适合丹东的质量更高、效益更好、结构更优的发展新路。

我们将坚持政治担当，树立正确的政绩观，摒弃盲目追求速度、有水分的增长，持之以恒地追求提质增效、结构优化。树立“功成不必在我任期”的理念，勇于承受改革的“代价”、创新的“风险”、转型的“阵痛”，守住经济运行、保障民生和社会稳定的底线，真正做到心无旁骛、保持战略定力。

我们将坚持务实高效的工作，切实转变政府职能和工作作风，千方百计提高效率、抓工作落实，深入调查研究，潜心解决难题，凡事定了就办，办就快办，办就办好。把加强软环境建设作为政府的硬抓手，开展领导干部和职能部门“不作为、不担当”问题专项整治，坚决查处为官不为、懒政怠政行为。

我们将坚持廉政为民，始终把为人民谋发展、增福祉作为最大责任，始终把实现丹东全面建成小康社会的使命扛在肩上，始终把群众冷暖忧乐放在心头。践行“三严三实”，切实落实“一岗双责”，坚持廉洁用权，倡俭治奢，深入推进党风廉政建设和反腐败工作，全面推动公务员队伍整体能力和水平的提升。

各位代表，我们已经走进了“十三五”，开启了新的征程。我们将在党的十八届三中、四中、五中全会精神指引下，在市委的正确领导下，在市人大和市政协的有力监督和支持下，进一步坚定信心，凝神聚力，开拓创新，奋发有为，全面完成各项工作目标任务，为推动全市经济社会平稳健康发展而努力奋斗!
